# Glutathione, carbohydrate and other metabolites of *Larix olgensis* A. Henry reponse to polyethylene glycol-simulated drought stress

**DOI:** 10.1371/journal.pone.0253780

**Published:** 2021-11-17

**Authors:** Lei Zhang, Shanshan Yan, Sufang Zhang, Pingyu Yan, Junhui Wang, Hanguo Zhang

**Affiliations:** 1 State Key Laboratory of Tree Genetics and Breeding, Northeast Forestry University, Harbin, China; 2 State Key Laboratory of Tree Genetics and Breeding, Chinese Academy of Forestry, Beijing, China; United Arab Emirates University, UNITED ARAB EMIRATES

## Abstract

Drought stress in trees limits their growth, survival, and productivity and it negatively affects the afforestation survival rate. Our study focused on the molecular responses to drought stress in a coniferous species *Larix olgensis* A. Henry. Drought stress was simulated in one-year-old seedlings using 25% polyethylene glycol 6000. The drought stress response in these seedlings was assessed by analyzing select biochemical parameters, along with gene expression and metabolite profiles. The soluble protein content, peroxidase activity, and malondialdehyde content of *L*. *olgensis* were significantly changed during drought stress. Quantitative gene expression analysis identified a total of 8172 differentially expressed genes in seedlings processed after 24 h, 48 h, and 96 h of drought stress treatment. Compared with the gene expression profile of the untreated control, the number of up-regulated genes was higher than that of down-regulated genes, indicating that *L*. *olgensis* mainly responded to drought stress through positive regulation. Metabolite analysis of the control and stress-treated samples showed that under drought stress, the increased abundance of linoleic acid was the highest among up-regulated metabolites, which also included some saccharides. A combined analysis of the transcriptome and metabolome revealed that genes dominating the differential expression profile were involved in glutathione metabolism, galactose metabolism, and starch and sucrose metabolism. Moreover, the relative abundance of specific metabolites of these pathways was also altered. Thus, our results indicated that *L*. *olgensis* prevented free radical-induced damage through glutathione metabolism and responded to drought through sugar accumulation.

## Introduction

Drought is a main limiting factor for tree growth, survival, and productivity, with a negative effect on the afforestation survival rate [1]. The response to drought stress is complex in the whole tree as well as at the tissue and cellular level. Drought resistance mechanisms vary among tree species with substantial differences between angiosperms (broad-leaved plants) and gymnosperms (coniferous plants). Molecular responses to drought stress have been extensively studied in broad-leaved species, but studies on coniferous species are limited [2]. *Larix olgensis* A. Henry is a more drought-tolerant conifer [3], which is mainly distributed in the Changbai Mountain and Laoyeling Mountain area of Northeast China. The lack of research on basic drought stress mechanisms in *L*. *olgensis* has hindered the improvement of larch yield and wood quality in arid environments to some extent. However, to potentially improve drought resistance in *L*. *olgensis*, it is necessary to explore drought stress-induced molecular mechanisms in this conifer.

With the rapid development of sequencing technology, molecular biology has entered the era of “big data” by facilitating research on physiological and biochemical changes in plants and animals through omics-based approaches, which provides explanations for some scientific problems at the genetic level that expand our understanding of certain physiological and biochemical processes. Specifically, transcriptome research can deepen the analysis of physiological processes based on the level of genes [4]. However, using a single-omics approach has failed to fully reveal the mechanism of the plant response to drought stress. Metabolomics is an emerging omics technique, adding a new level to the established approaches in genomics, proteomics, and transcriptomics. Data sets generated by measuring changes in the gene expression and metabolite levels in the transcriptome and metabolome, respectively, can be used for association analyses. This approach has been utilized in drought resistance studies in plants, such as ryegrass [5], poplar [6], *Astragalus membranaceus* [7], and *Panicum virgatum L*. [8]. However, there are no relevant reports on conifers.

To maintain normal growth and development under drought stress, plants respond by activating a series of physiological and biochemical regulatory mechanisms [9]. The drought stress response is a complex biochemical process that diminishes the effects of drought through molecular mechanisms that ensure osmotic adjustment, along with proper levels of antioxidants and scavenger compounds.

Under drought stress, plants produce many changes such as physiology and growth characteristics [10], gene expression [11] and metabolites [12] to maintain the osmotic pressure and balance, thus, maintaining the normal physiological and biochemical processes and preventing drought stress-induced damage. Under drought stress, plants regulate the expression of many synthetase genes, leading to increased concentrations of soluble metabolites, such as free proline, soluble sugars, organic acids, and betaine [13]. Osmotic regulation by accumulating solutes is a main mechanism in plants to adapt to drought stress. During drought, these compounds maintain the osmotic balance between the cytoplasmic matrix and the environment by preventing water loss and protecting membrane integrity [14]. Similar to other stress conditions, such as high temperature, drought stress is accompanied by increased reactive oxygen species (ROS) production. Plants prevent ROS production and oxidative stress through complex antioxidant defense systems involving multiple enzymes and antioxidants. The main non-enzymatic antioxidants are L-ascorbic acid (AsA) and glutathione (GSH), and critical enzymes are glutathione reductase (GR), glutathione peroxidase (GPX), and glutathione S-transferase (GST) [15]. Thus, combining transcriptomics and metabolomics methods should generate results that will expand our knowledge of the mechanism of drought stress resistance in plants.

In recent years, “spring drought” has frequently occurred in the northeastern part of China, which has severely slowed the growth and development of forest trees. As a native tree species in Northeast China, *L*. *olgensis* has evolved drought resistance through “spring drought” cycles. However, the response mechanisms against drought stress in *L*. *olgensis* are still unclear. By performing a combined analysis of the physiological, gene expression, and metabolite profile changes under drought stress, we aimed to identify the main metabolic pathways for regulating the responses to drought stress, which were critical for studying the mechanism of the drought resistance in *L*. *olgensis*.

## Results

### Analysis of biochemical changes under PEG-simulated drought stress

**Fig 1:**
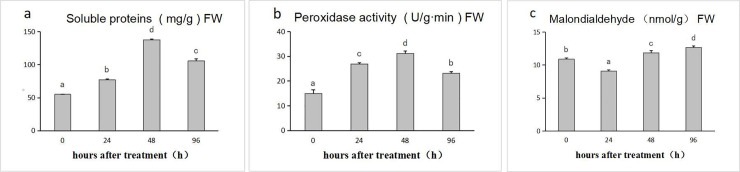
Physiological and biochemical changes in *Larix olgensis* under drought stress. (a) Effect of drought stress on soluble protein (SP) content. (b) Effect of drought stress on peroxidase (POD) activity. (c) Effect of drought stress on malondialdehyde (MDA) content. Error bars represent standard error.

During drought stress in *L*. *olgensis*, the SP content generally increased. In the early stage of drought stress (0–24 h), the SP content increased from 55 to 77 mg g^-1^. During drought stress from 24 h to 48 h, the SP content reached a peak value of 137 mg g^-1^ (Fig 1A).

In the early stage of drought stress, POD activity increased from 15.0 to 26.8 U min^-1^ g^-1^. During drought stress, POD activity increased to 31.2 U min^-1^ g^-1^ at 48 h, reaching a peak value, and subsequently decreasing to 23.2 U min^-1^ g^-1^ at 96 h. Over the whole drought stress period, the POD activity showed a “rise-rise-decline” change, but the overall POD activity showed an upward trend (Fig 1B).

The MDA content increased from 10.9 to 12.7 nmol g^-1^, displaying a “decline-rise” trend over the whole drought stress period (Fig 1C). Thus, the monitoring of select biochemical parameters indicated that the SP content, MDA content, and POD activity varied significantly throughout the consecutive drought stress periods, revealing some changes in *L*. *olgensis* associated with drought stress, which were related to gene expression levels and variations in metabolite content, indicating the need to conduct a transcriptomics and metabolomics analysis.

### Transcriptomic analysis of *L*. *olgensis* under drought stress

The cDNA library of drought stress-treated *L*. *olgensis* was sequenced using an Illumina Hiseq high-throughput sequencing platform based on sequencing by synthesis. The transcriptome sequencing of all samples yielded a clean data set of 36.88 Gb. The high-quality sequence data were assembled using the Trinity software. A total of 59 710 unigenes with an N50 of 1709 bp were obtained. Their assembly integrity was high; 21 907 unigenes had a length of ≥1000 bp.

### Functional analysis of DEGs

**Fig 2:**
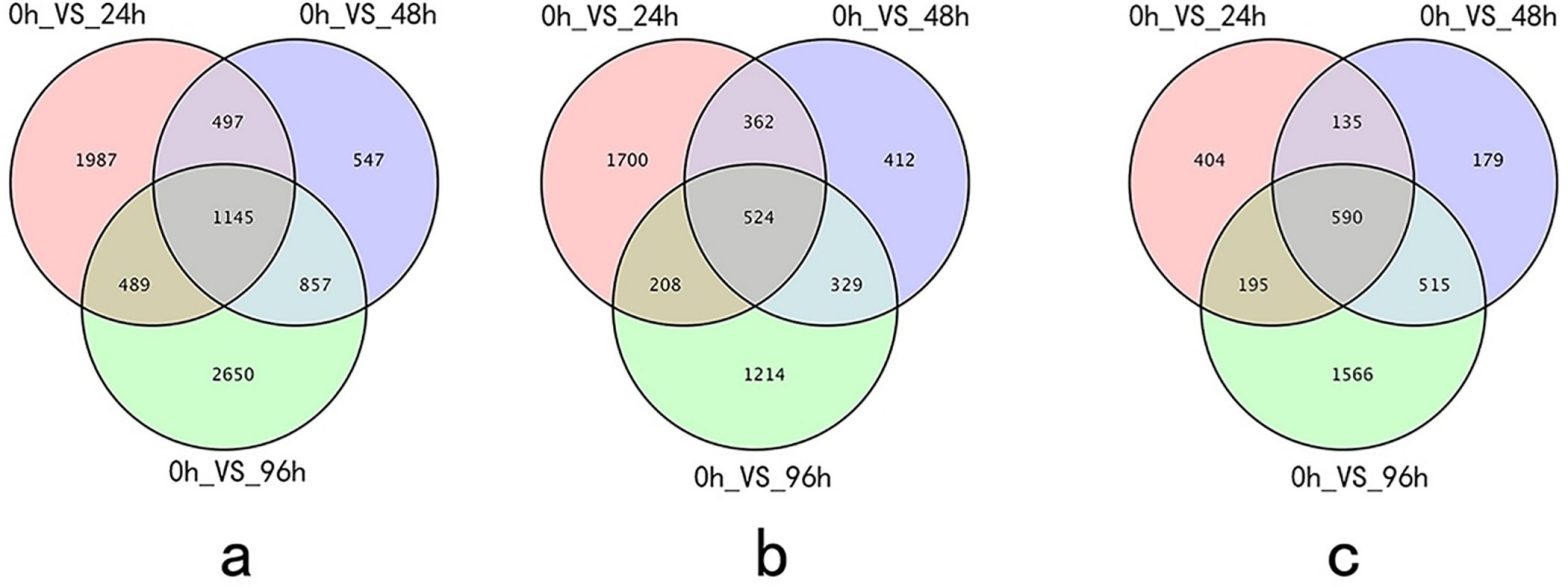
Drought-induced, differentially expressed genes in *Larix olgensis*. (a) Venn map of differentially expressed genes after 24 h, 48 h, and 96 h under drought stress; (b) Venn map of genes that were only up-regulated after 24 h, 48 h, and 96 h under drought stress; (c) Venn map of genes that were only down-regulated after 24 h, 48 h, and 96 h under drought stress.

The total number of DEGs was 8172 in the drought treatment samples collected after 24 h, 48 h, and 96 h. Compared with the gene expression level in the control (0 h), the drought treatment samples collected after 24 h, 48 h, and 96 h had 4118 DEGs (up-regulated: 2794; down-regulated: 1224), 3046 DEGs (up-regulated: 1627; down-regulated: 1419), and 5141 DEGs (up-regulated: 2275; down-regulated: 2866), respectively (Table 1). A total of 1145 DEGs were co-expressed during all drought stress periods; 524 DEGs were only significantly up-regulated, and 590 DEGs were only down-regulated (Fig 2).

**Table 1:** The number of differentially expressed genes.

DEG_Set	All_DEG	Up regulated	Down regulated
T01_vs_T02	4118	2794	1324
T01_vs_T03	3046	1627	1419
T01_vs_T04	5141	2275	2886

T01: Stress treatment 0 h, T02: Stress treatment 24 h, T03: Stress treatment 48 h, T04: Stress treatment 96 h.

**Fig 3:**
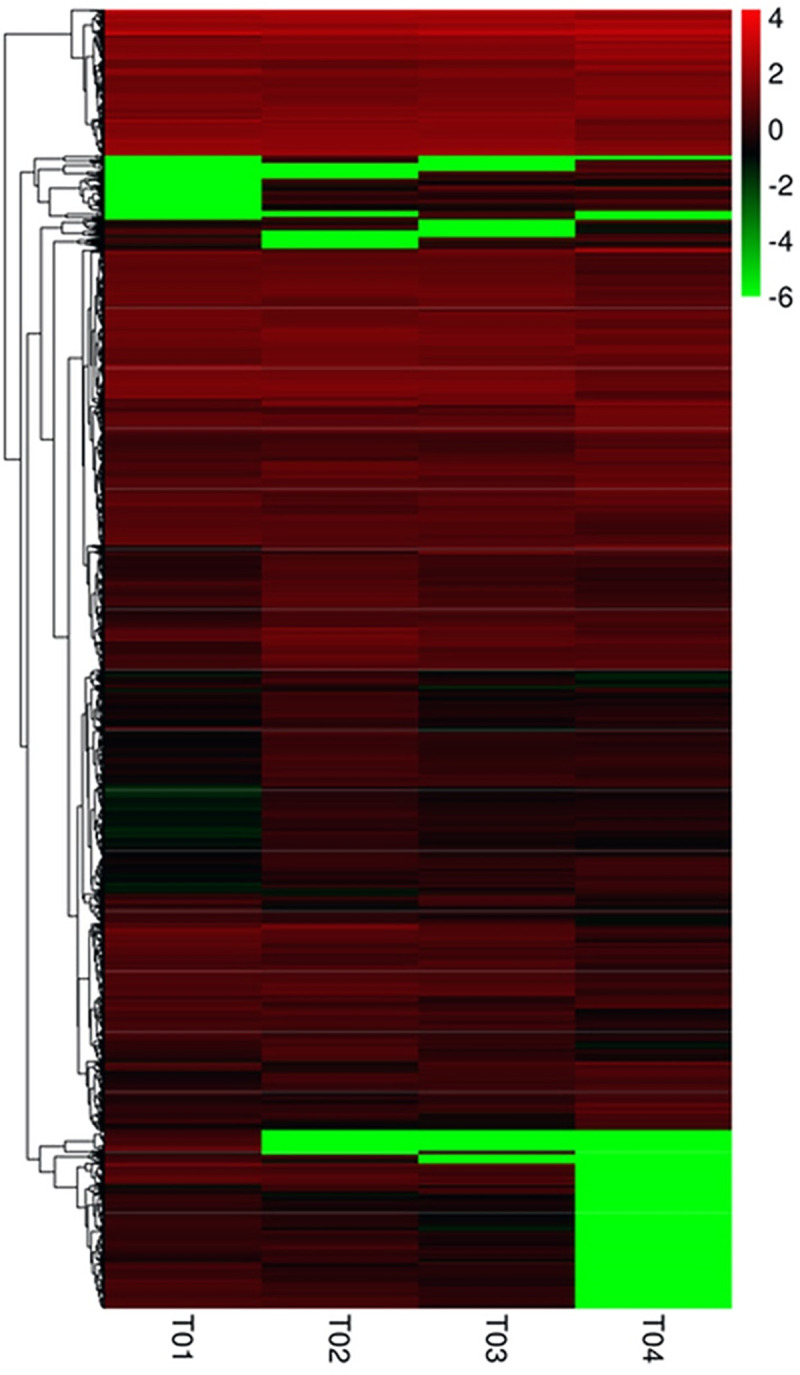
Differentially expressed genes cluster. Columns represent different samples; rows are different genes. The green color indicates the down-regulated and red indicates the up-regulated expression of genes. T01: Stress treatment 0 h, T02: Stress treatment 24 h, T03: Stress treatment 48 h, T04: Stress treatment 96 h.

The complete transcriptional profile of co-expressed transcripts at different stages of drought stress was obtained by hierarchical clustering. Clustering spectra indicated that drought stress significantly affected the transcriptional profile of the co-expressed transcripts. Compared with the control expression levels, there were more up-regulated genes than down-regulated genes among the co-expressed transcripts (Fig 3).

**Fig 4:**
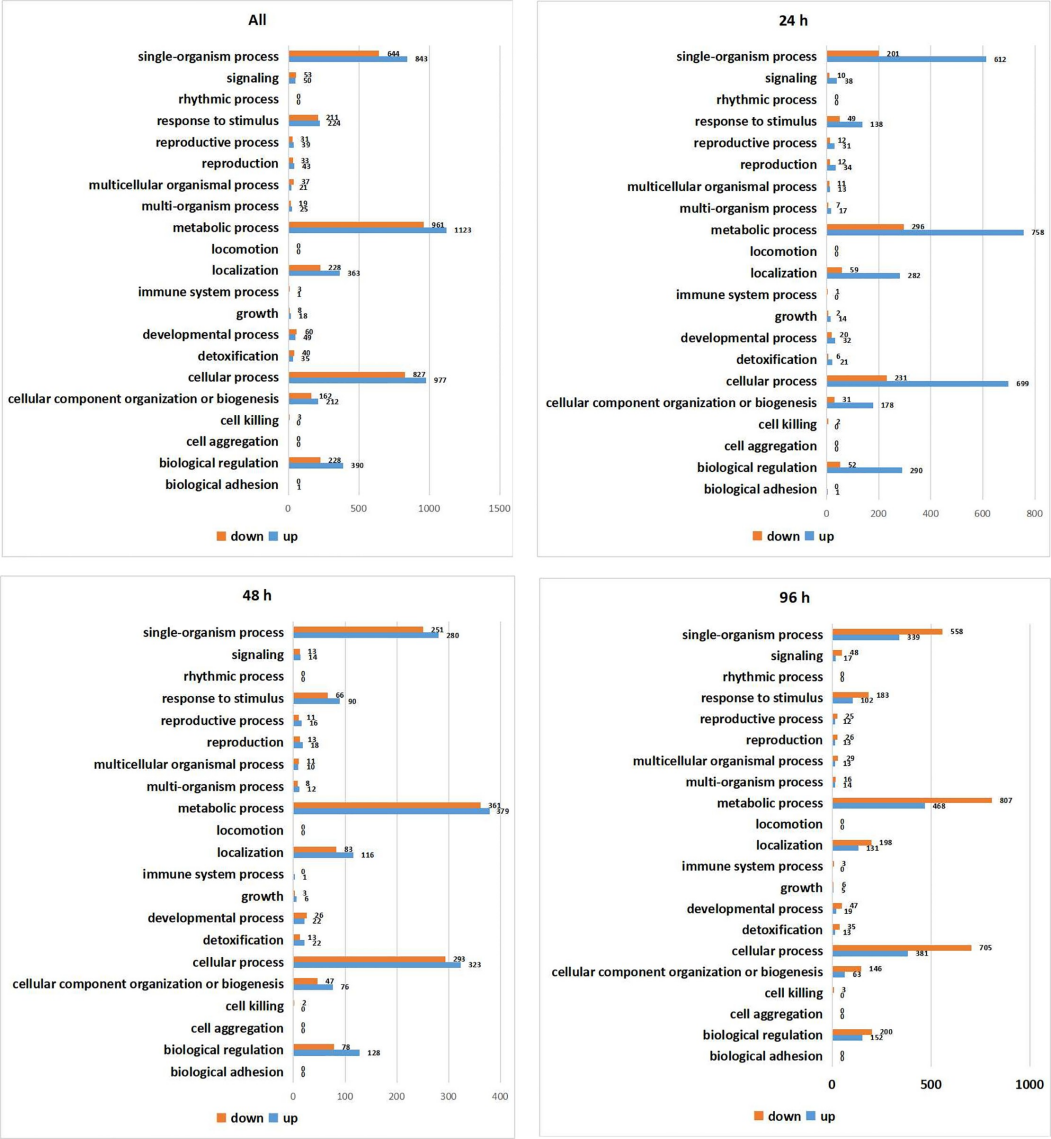
Plots of gene ontology (GO) terms derived from the biological process category of the genes. (a) DEGs in total. (b) DEGs under drought treatment for 24 h (compared to the control). (c) DEGs under drought treatment for 48 h (compared to the control). (d) DEGs under drought treatment for 96 h (compared to the control).

GO is an internationally standardized gene function classification system with three main GO categories, cellular component, molecular function, and biological process. Among these categories, the DEGs were enriched under the GO term metabolic process, followed by the terms cellular process and single-organism process (Fig 4A). After 24 h of drought stress, metabolic, cellular, and single-organism processes were associated with more up-regulated than down-regulated DEGs (Fig 4B), but after 48 h of drought stress, the same GO process terms had more down-regulated DEGs (Fig 4B and 4C). After 96 h of drought stress, metabolic, cellular, and single-organism processes were more abundant among down-regulated DEGs than among up-regulated DEGs (Fig 4B and 4D). After 24 h of drought stress, 52 DEGs were enriched in biological regulation, which increased to 200 DEGs after 96 h of drought stress.

### Gene expression response to drought

Based on the gene expression ratio between 24 h drought stress and the control, an analysis of the 50 genes with the highest differential expression found that a gene annotated with the Jacalin-like lectin domain had the highest expression, and there were a total of three annotations for the Jacalin-like lectin domain and one annotation for peroxidase among the top 50 DEGs. After 48 h of drought stress, the top 50 DEGs included two genes with an annotation for heat shock protein (HSP), and two other genes were associated with ATP. Among the top 50 DEGs at 96 h of drought stress, the highest expressed gene annotation was a bidirectional sugar transporter, and three genes had the annotation HSP20/α crystallin family; two other genes were annotated as late embryogenesis abundant (LEA) genes (Supporting information: The top 50 differentially expressed genes and their predicted functions.).

### DEGs during drought stress

Non-specific lipid transfer proteins (nsLTPs) are a class of small, basic proteins in plants. Our results showed that three genes annotated as nsLTPs were among the DEGs that were up-regulated throughout three drought stress periods (Table 2). LEA proteins are small, hydrophilic proteins with a protective function associated with water deficit. A total of 11 drought stress-associated DEGs were annotated as LEA protein genes, which were largely expressed during the last drought stress period, indicating that they are late response proteins during drought stress in *L*. *olgensis* (Table 3).

**Table 2:** Response of the non-specific lipid transfer protein genes to drought stress.

Gene ID	log_2_FC		
	0vs24	0vs48	0vs96
c78994.graph_c0	1.74	2.01	2.16
c115547.graph_c1	1.74	1.31	1.04
c120264.graph_c0	0.98	1.84	4.49

**Table 3:** Response of LEA genes to drought stress.

Gene ID	log_2_FC		
	0vs24	0vs48	0vs96
c76376.graph_c0	--	1.72	5.72
c118163.graph_c0	--	7.37	7.93
c115421.graph_c1	-0.04	2.35	5.96
c112578.graph_c0	0.10	1.22	4.60
c81527.graph_c0	--	--	4.98
c113561.graph_c0	-0.74	-0.29	3.78
c87621.graph_c0	--	--	8.70
c111067.graph_c0	--	--	8.01
c111234.graph_c0	--	--	7.00
c117289.graph_c0	1.91	1.40	4.92
c101723.graph_c0	-0.99	0.35	3.92

### Metabolic changes in *L*. *olgensis* during drought stress

**Fig 5:**
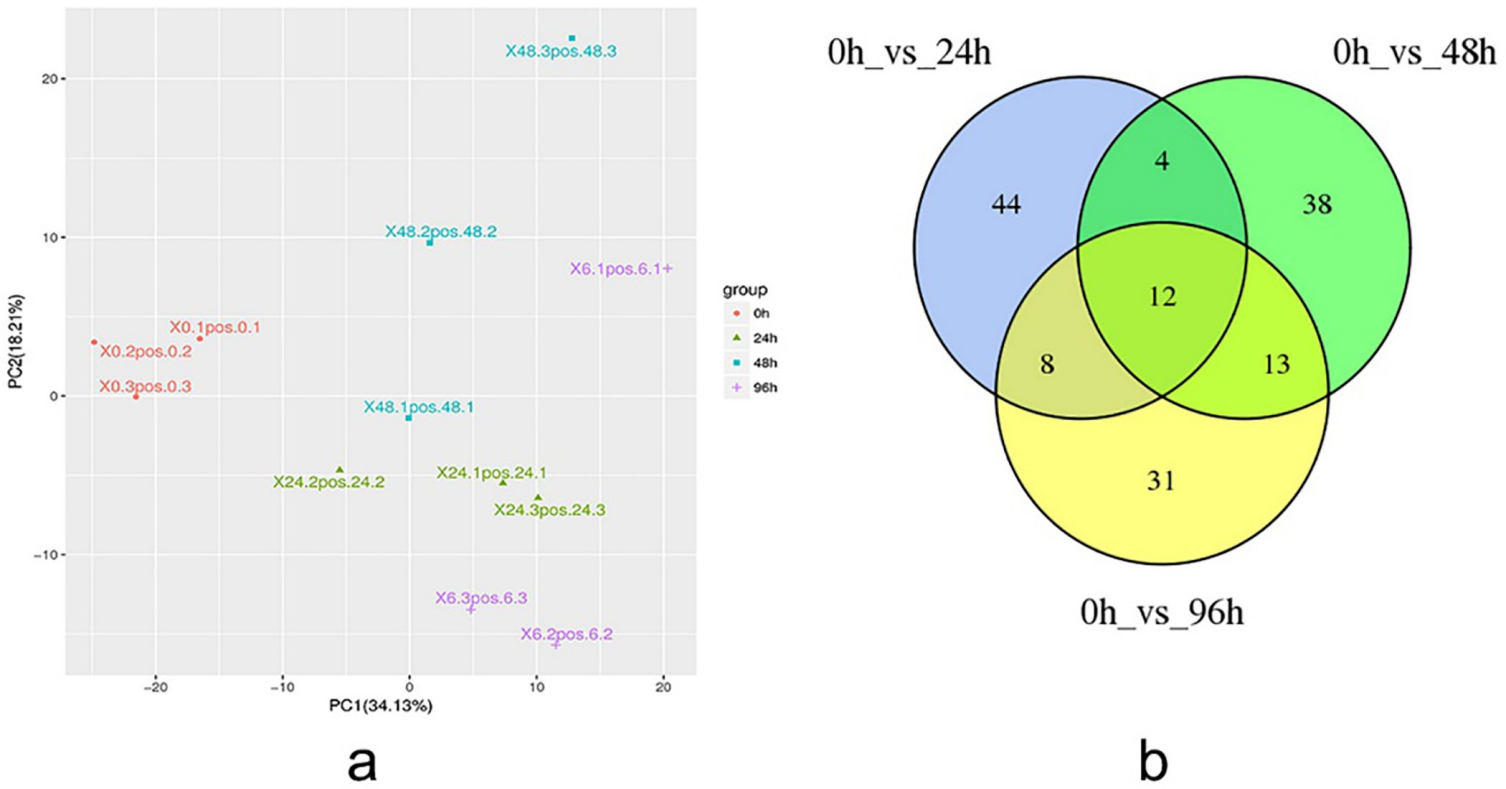
Metabolite analysis. (a) Sample principal component analysis chart. (b) Venn diagram of differential metabolites from each drought treatment period.

Samples were evaluated for repeated biological correlations using the Spearman rank correlation. Repeated correlations between samples were above 0.7, indicating that the entire metabolome assay was reliable. Prior to the difference analysis, the different groups were subjected to a Principal component analysis (PCA). Twelve samples were divided into four components by principal component PC1 (34.13%) and principal component PC2 (18.21%), wherein the main component PC1 clearly separated the control group from each treatment group, indicating a large difference between the treatment and control after drought stress treatment (Fig 5).

The screen for differential metabolites was performed using a combination of the *P* value from Student’s *t*-test and the variable influence on projection (VIP) value from the orthogonal projections to latent structures discriminant analysis (OPLS-DA) model. The screening criteria were *P*<0.05 and VIP>1. After 24 h of drought stress, 11 differentially expressed metabolites were increased, and after 96 h of drought stress, there was an increase in 42 differentially expressed metabolites (Table 4). A total of 12 metabolites were differentially expressed throughout the entire drought stress treatment (Fig 5).

**Table 4:** Differentially expressed metabolites.

Group name	All diff	up-regulated	down-regulated
T01_vs_T02	58	20	38
T01_vs_T03	33	11	22
T01_vs_T04	115	42	73

The level of metabolite expression changed in a time-dependent manner throughout the 96 h of drought stress treatment. Specifically, among the differentially expressed metabolites, linoleic acid and d-imidazoleglycerol-phosphate dehydratase had the most pronounced change in expression level compared with that in the control. Under long-term drought stress, the abundance of linoleic acid and d-imidazoleglycerol-phosphate dehydratase increased 5.36 times and 4.63 times, respectively. During the drought stress treatment, the abundance of galactose, maltose, mannose, raffinose, D-glucose 6-phosphate, and inositol was increased after 24 h, slightly reduced after 48 h, and increased again after long-term drought stress that lasted for 96 h. The expression of some alkaloids, such as betaine, acetylcholine, and glycerophosphocholine, was continuously up-regulated in a time-dependent manner during drought stress. Most amino acids, such as isoleucine, arginine, lysine, and glutamic acid, were initially up-regulated in the early drought stress period but gradually decreased during the remainder of the drought stress treatment. However, the level of proline was elevated throughout the whole drought stress treatment, compared with that in the control, and the maximum level was reached after 96 h long-term drought treatment (Table 5).

**Table 5:** Expression of differentially expressed metabolites.

Metabolites	log_2_FC		
	0vs24	0vs48	0vs96
L-valine	0.72	0.10	2.70
Isoleucine, arginine	0.24	-1.10	-0.50
Lysine-PRO	0.66	-0.19	-0.33
Isoleucine, glutamic acid	0.50	0.01	-0.79
Betaine	0.64	0.41	0.22
Acetylcholine	1.37	0.42	0.89
Glycerol phosphate	1.32	0.64	0.63
L-fucose	0.59	-0.18	0.08
Raffinose	1.25	-0.14	1.76
d mannose	0.74	-0.43	0.90
D-glucose 6-phosphate	0.71	-0.21	0.55
Galactoside	0.53	-0.26	1.04
Maltose	0.289	-0.15	0.20
D-erythridazole glycerol phosphate	3.92	3.59	4.63
Linoleic acid	4.60	4.32	5.36
Inositol	2.11	-0.63	1.59
Fenugreek	3.26	3.54	4.46

### Comprehensive analysis of gene expression and metabolite levels

**Fig 6:**
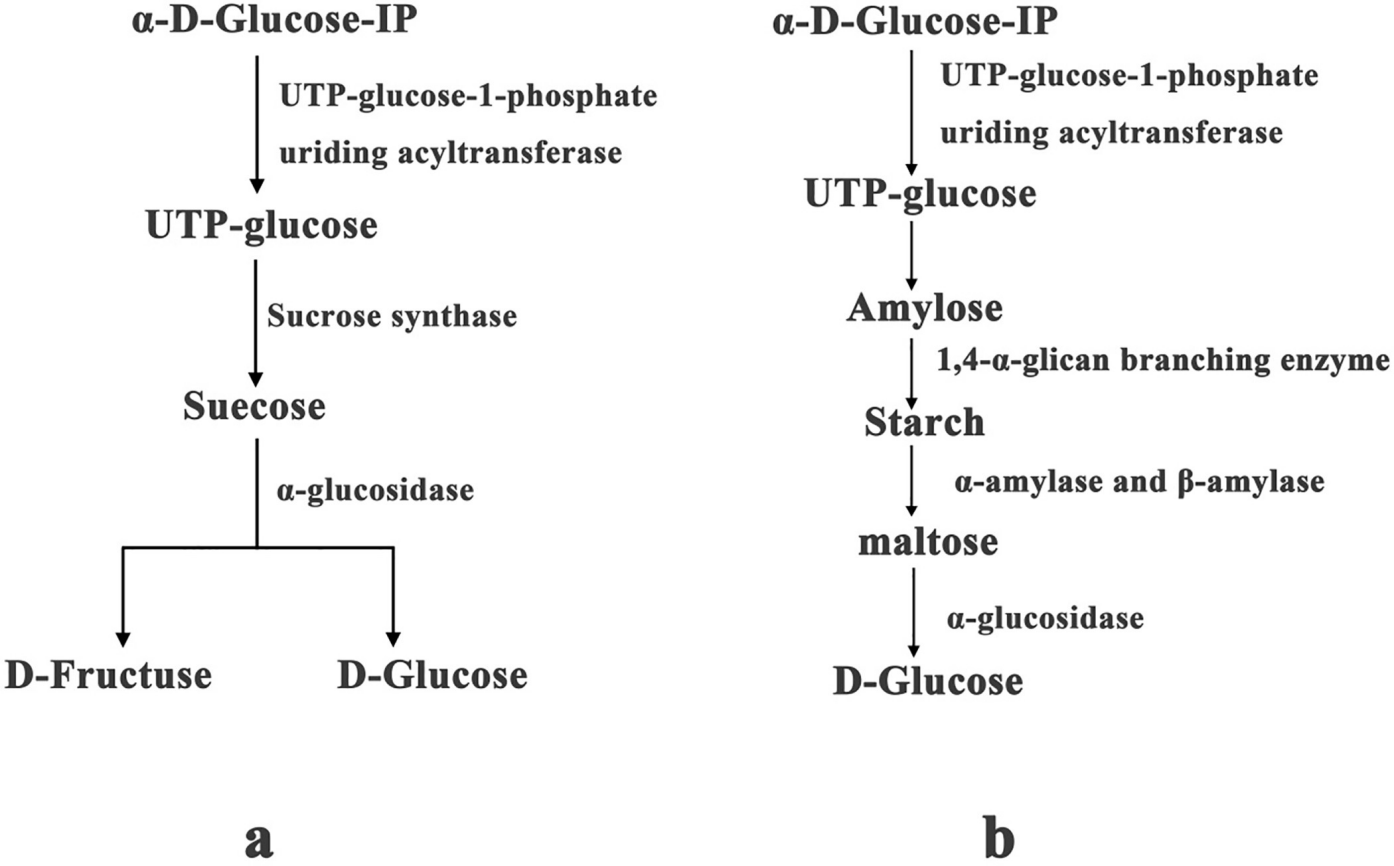
Changes in leaf starch and sucrose pathway gene expression under drought stress. Altered expression of genes involved in sucrose metabolism (a) and starch metabolism (b).

**Fig 7:**
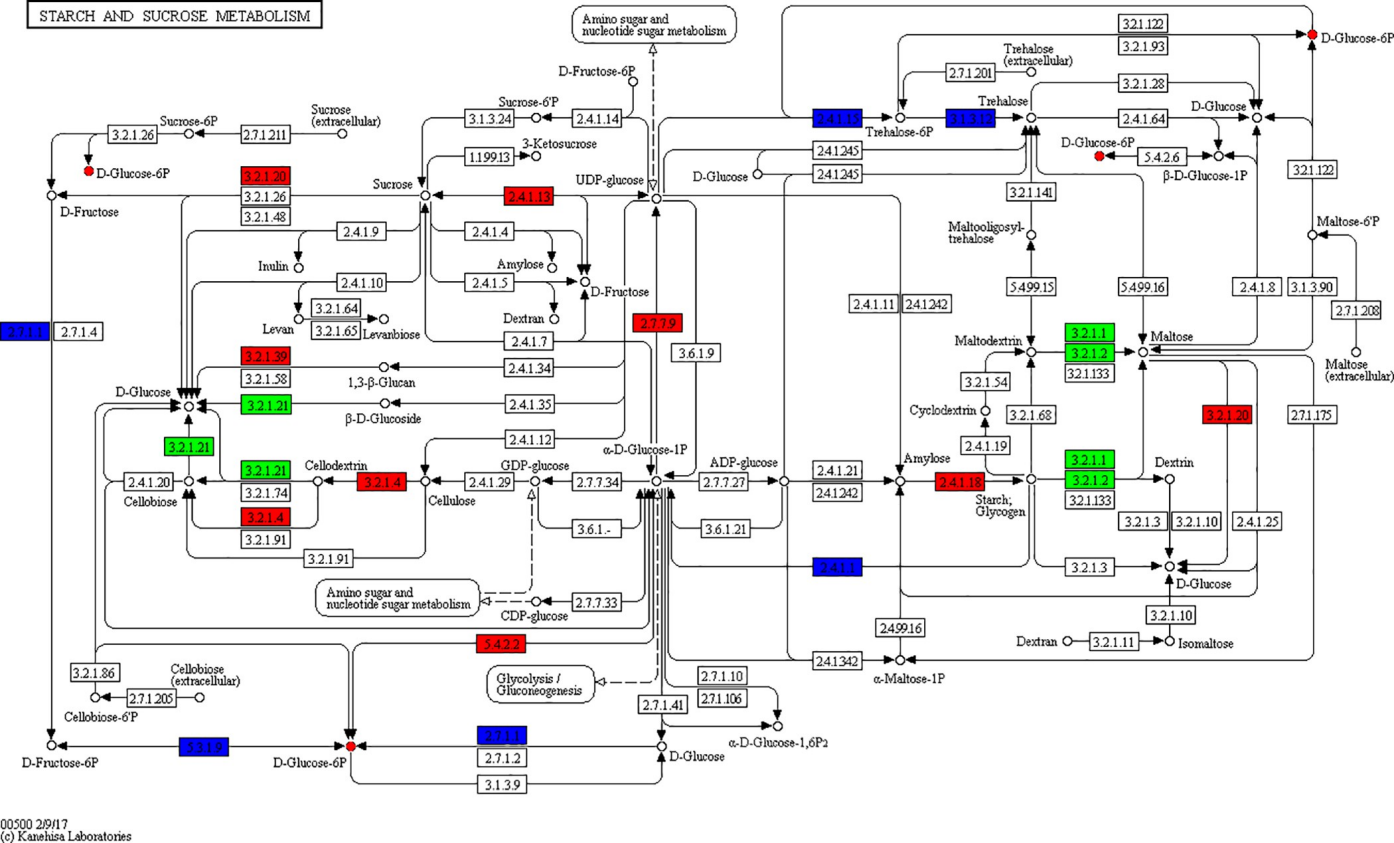
Starch and sucrose metabolism pathway.

To better assess transcriptional regulation mechanisms in *L*. *olgensis* under drought stress, a correlation analysis was performed between the metabolome and the transcriptome data sets. Using the analysis results for differential metabolites and transcriptome-based differential genes, the same groups of differential metabolites and genes were simultaneously mapped on the KEGG pathway map, and 20 KEGG pathways were obtained. The results indicated that drought stress significantly affected fructose and mannose metabolism, galactose metabolism, biosynthesis of keratin, berberine, and wax, starch and sucrose metabolism, amino acid sugar and nucleotide sugar metabolism, inositol phosphate metabolism, glyceryl phosphatide metabolism, and GSH metabolism. The relationship between metabolite abundance and gene expression in GSH metabolism, galactose metabolism, and starch and sucrose metabolism was determined (Tables 6 and 7) (Figs 6 and 7).

**Table 6:** Annotations of differentially expressed genes in response to drought in glutathione metabolites of *Larix olgensis*.

Pathway	Glutathione metabolism			
Metabolites	5-L glutamyl-L-alanine			
Gene ID	Gene annotation	log_2_FC		
		0vs24	0vs48	0vs96
c80872.graph_c0	glucose-6-phosphate 1-dehydrogenase	2.77	1.59	0.86
c129125.graph_c0	glucose-6-phosphate 1-dehydrogenase	3.76	5.56	5.23
c121889.graph_c0	glucose-6-phosphate 1-dehydrogenase	-0.74	-0.01	-0.41
c126867.graph_c0	glucose-6-phosphate 1-dehydrogenase	-0.48	0.09	-0.51
c119110.graph_c0	6-phosphogluconate dehydrogenase	0.39	0.20	0.39
c119707.graph_c0	6-phosphogluconate dehydrogenase	0.60	1.17	6.39
c104199.graph_c0	6-phosphogluconate dehydrogenase	1.28	2.08	2.87
c127402.graph_c0	glutathione peroxidase	1.76	2.35	2.11
c104048.graph_c0	glutathione peroxidase	0.65	3.02	5.03
c116017.graph_c0	glutathione reductase	0.40	0.19	0.95
c127613.graph_c0	glutathione reductase	3.02	1.63	1.59
c117740.graph_c2	glutathione S-transferase	1.62	2.62	4.00
c111760.graph_c0	glutathione S-transferase	--	3.06	2.47
c109099.graph_c1	glutathione S-transferase	0.90	1.17	0.77
c107334.graph_c0	glutathione S-transferase	0.98	1.52	2.38

**Table 7:** Annotation of differentially expressed genes in response to drought in galactose metabolites of *Larix olgensis*.

Pathway	Galactose metabolism			
Metabolites	Raffinose, Inositol galactose, D-glucose and D-fructose			
Gene ID	Gene annotation	log_2_FC		
		0vs24	0vs48	0vs96
c78402.graph_c0	UDP-glucose 4-epimerase	4.37	3.20	3.30
c105959.graph_c0	UDP glucose-hexose-1-phosphate uridine acyltransferase	2.75	--	--
c68977.graph_c0	UTP-glucose-1-phosphate uridine acyltransferase	2.04	-0.34	-0.40
c67404.graph_c0	Hexokinase	2.37	--	--
c66344.graph_c0	Hexokinase	2.47	1.19	0.96
c114704.graph_c0	Hexokinase	-1.74	-1.78	-5.99
c127353.graph_c0	Glucosidase	2.45	-0.84	-1.79
c126590.graph_c0	Inositol 3-α-galactosyltransferase	1.41	0.01	2.09
c113769.graph_c0	Inositol 3-α-galactosyltransferase	1.29	0.37	0.38
c122961.graph_c1	Stachyose synthase	-1.14	-0.57	-1.06
c113459.graph_c1	Alpha-galactosidase	-1.37	-0.07	-1.20
c111540.graph_c0	Alpha-glucosidase	2.74	1.60	--
c74583.graph_c1	Beta-galactosidase	-5.25	-5.52	-5.19
c74583.graph_c0	Beta-galactosidase	-4.65	-4.91	-4.59
c102649.graph_c0	6-phosphofructokinase 1	2.80	1.77	2.34
c120102.graph_c0	Raffinose synthase	0.32	0.54	2.35
c114864.graph_c0	Raffinose synthase	1.37	--	2.72
c119962.graph_c4	Raffinose synthase	2.27	1.67	2.82
c108549.graph_c0	Raffinose synthase	0.81	0.69	2.99
c84960.graph_c0	Raffinose synthase	1.66	1.31	--

The GSH metabolic pathway had two annotated GPX and two annotated GR genes, all of which were up-regulated during drought stress, compared with the control transcription profile. From the four genes annotated as glucose-6-phosphate 1-dehydrogenase, two were up-regulated, but the other two were down-regulated during drought stress. Three genes were annotated as 6-phosphogluconate dehydrogenase, and two enzymes were involved in the conversion of NADP/NADPH. There were four genes annotated as glutathione S-transferase, which showed up-regulated expression under drought stress. One gene was annotated as L-ascorbate peroxidase, and in addition, one gene was annotated as leucyl aminopeptidase, which was involved in cysteine and glycine synthesis.

In galactose metabolism (Table 7), the abundance of several metabolites, raffinose, inositol galactose, D-glucose, and D-fructose, were increased. A total of 114 genes were obtained by analyzing galactose metabolism-related genes, and the respective gene annotations identified 12 enzymes. Two annotated inositol 3-α-galactosyltransferase genes were up-regulated during the three drought stress periods. There were six annotated raffinose synthase genes and one annotated stachyose synthetase gene, the latter being a key enzyme in stachyose synthesis. These genes were also up-regulated during drought stress. One gene was annotated as α-glucosidase, and two genes were annotated as α-galactosidase. The changes in the expression level of these genes increased the raffinose content by 1.24 times during the drought.

In sucrose metabolism (Fig 6A), the gene encoding sucrose synthase was up-regulated after 24 h and 48 h of drought stress but slightly down-regulated under long-term drought stress lasting for 96 h. The α-glucosidase gene was up-regulated throughout the drought stress treatment, and the UTP-glucose-1-phosphate uridine acyltransferase gene was up-regulated during the early drought period. Compared with the sucrose profile in the control, the sucrose content was significantly increased during the early drought stress period but decreased after the long-term treatment, which may be related to the large amount of sucrose decomposed to D-fructose during the last drought stress period; specifically, the D-fructose content (1.18620722, 0.245392272, 1.45427517) was increased relative to that in the control.

In starch metabolism (Fig 6B), the glucose content (2.416205353, 2.180226375, 2.741899739) was increased by the drought stress treatment. The β-amylase and α-amylase genes were down-regulated after 24 h of drought stress but up-regulated after 48 h and 96 h of drought stress. Additionally, the 1,4-α-glucan branching enzyme gene was up-regulated during the initial 24 h of drought stress but down-regulated after the 48 h and 96 h drought stress periods. The expression level changes in these genes caused starch accumulation during the early drought stress period compared with the starch content in the control. Moreover, the α-glucosidase gene was up-regulated during the last drought stress period.

## Discussion

Select biochemical parameters of *L*. *olgensis* were determined under PEG-simulated drought stress before treatment initiation (0 h) and after 24 h, 48 h, and 96 h. Based on transcriptomics and metabolomics data, the effects of drought stress were studied, and the metabolic pathways and related genes that changed during the drought stress treatment were analyzed.

Under normal environmental conditions, the metabolic, physiological, and biochemical processes in plants are relatively stable; however, various metabolic activities in the plant can change in response to adverse conditions and stress. Osmotic adjustment is a critical physiological mechanism for plants to endure and resist drought and an important physiological indicator for selecting drought-tolerant crops. Osmotic adjustment typically occurs when the degree of drought stress is mild or moderate. During environmental stress, various organic and inorganic substances accumulate in the plant, increasing their cytoplasmic concentration and reducing the osmotic potential, all of which maintain the water balance in the plant for adaptation to the adverse environment. When the degree of water stress is severe, the osmotic adjustment ability is weakened or lost. Under PEG stress, changes in the SP content showed a “increase-decrease” trend over the entire stress period. The SP content (106.726 mg/g) after 96 h of PEG stress treatment was 93.61% higher than that of the control (55.125 mg/g), indicating that the plant underwent a strong osmotic adjustment during PEG stress treatment.

Plants produce low concentrations of ROS under normal growth conditions. However, under drought stress, ROS production is increased, which causes oxidative damage in plants [16]. Plants act through an enzymatic protection system that uses POD to scavenge ROS, thereby protecting the membrane system from damage [17]. The POD activity (23.2) after 96 h of PEG stress treatment was 54.67% higher than that of the control (15.0), and the overall trend was upward.

In arid environments, cell membrane permeability changes, resulting in an increase in relative conductivity and triggering membrane lipid peroxidation, which generates MDA, causing damage to cells due to increased MDA content [18]. Therefore, the MDA content is an indicator for the degree of damage to the plant under drought stress, which indirectly reflects the plant’s drought resistance; that is, the higher the MDA content, the greater the plant damage and the worse the drought resistance [19]. The MDA content (12.67 nmol·g^-1^) under stress treatment for 96 h was increased by 18.05% compared with that in the control (10.73 nmol·g^-1^), it showed the balance between the production and scavenging of reactive oxygen species in plant cells is destroyed, resulting in the enrichment of reactive oxygen species in large amounts, resulting in the peroxidation of unsaturated bonds in fatty acids in the cell intima. The MDA content was decreased after 24 h of PEG stress treatment, maybe cause by the increase of POD activity is due to the increase of oxygen ion free radicals in plant somatic cells due to drought stress. In order to maintain the stability of plant cell membrane and prevent plants from being seriously damaged, the protective enzyme activity increases rapidly to remove excess oxygen ion free radicals, but the increase in the POD content to remove a portion of MDA during the initial 24 h. However, with the prolongation of stress time, the regulation ability decreased, and the MDA content continued to increase.

Due to the significant differences in biochemical indicators between the stress periods, the same four treatment periods were used for analyzing the DEGs and metabolites. The total number of drought-responsive genes was related to the length of the drought stress period. Specifically, the total number of DEGs after 24 h of stress treatment was lower than that after 96 h. HSPs are responsible for protein folding, assembly, and translocation, and the degradation of damaged proteins. They play a key role in plants, conferring protection against stress by stabilizing proteins and membranes [20]. During long-time drought stress, several genes related to HSPs were up-regulated, such as LEA proteins genes (8.69 times higher than the control value at 0 h). The LEA genes encode hydrophilic proteins that strongly bind to water, thus retaining moisture, preventing the crystallization of important cellular proteins and other molecules in the absence of water, and stabilizing the cell membrane [21]. In addition, the expression of genes annotated with the Jacalin-like lectin domain was up-regulated. Plant lectins, which reversibly bind to carbohydrates, are associated with plant stress resistance [22].

Similar to the DEGs analysis, we identified many metabolites associated with drought stress. Trigonelline (TG) is an alkaloid found in the leguminous plant *Trigonella foenum-graecum* (Fabaceae). As a penetrant, its accumulation reduces the osmotic potential of cells. Thus, TG plays an important role in protecting the cell membrane stability in plants under environmental stress [23]. In our study, TG accumulated 4.46 times in *L*. *ogensis* seedlings due to drought stress. Proline is widely recognized as a critical drought-induced metabolite that protects cell membranes by reducing the cell osmotic potential. In addition, it is also a regulator of the cellular redox state and an ROS scavenger [24]. The results showed that proline was only up-regulated after long-term drought stress, with an upregulation of 2.69 times. Under drought stress, soluble sugar usually accumulates and functions as a signal and osmotic adjustment [25]. The content of monosaccharides (glucose and fructose) also increased in this study, and the genes involved in the decomposition of polysaccharides were up-regulated.

After a comprehensive analysis of the metabolomics and transcriptome data, we obtained more information about metabolic pathways. Among the DEGs and metabolites, GSH and soluble sugars have significant regulatory properties (Table 5); further analysis focused on GSH metabolism, galactose metabolism, and sucrose and starch metabolism pathways.

GSH, a tripeptide widely present in organisms, has important physiological functions [26]. The active sulfhydryl group in the GSH structure binds to free radicals, which plays an important role in the cellular capacity for scavenging ROS. In this process, GST plays an important catalytic role. GST is mainly present in the cytoplasm and functions in oxidative stress resistance under biotic or abiotic stress. GST acts as a detoxifying agent for some extracellular substances that are harmful in cells, and it is also known to inhibit lipid peroxidation *in vitro* [27]. In our study, four GST-related genes were up-regulated to different degrees, indicating that GST was actively involved in the drought stress response and helped to maintain normal growth and development of the plants.

As the most abundant antioxidant in the cell, GSH protects DNA, proteins, and other biomolecules against oxidative damage and converts ROS and free radicals in general into metabolites that are easily eliminated *in vivo* [28]. Reduced GSH can reduce H_2_O_2_ to H_2_O by GPX in the process of scavenging ROS, and GSH itself is oxidized to glutathione disulfide (GSSG), while GSSG is mainly re-reduced to GSH by GR [29]. Through the conversion between reduced and oxidized forms, active oxygen is continuously removed, and the effect of ROS on the plant is reduced [30]. Our transcriptome analysis identified two genes annotated as GPX and one annotated as GR, all of which were up-regulated during drought stress. This result indicated that these two key enzymes actively respond to drought stress and play an important role in the process of scavenging ROS. Glucose-6-phosphate 1-dehydrogenase and 6-phosphogluconate dehydrogenase (Table 6, Fig 6) participate in the conversion of NADP/NADPH and use NADP as an electron acceptor to catalyze the formation of NADPH, which provides hydrogen reduction during the conversion of oxidized GSSG to reduced GSH [31]. The genes involved in the regulation of these enzymes were also up-regulated in our study, indicating that under the drought stress-induced action of reductase, the coenzyme with hydrogen reduction activity also responds positively to drought stress.

AsA, as an important antioxidant in cells, plays an important role in scavenging ROS. In addition, AsA can act as a cofactor for some antioxidant enzymes under stress [32]. Under drought stress, the AsA content increased significantly, and the ribonucleoside-diphosphate reductase associated with ascorbate synthesis was up-regulated, and the ascorbate reductase-related gene associated with AsA-mediated scavenging of hydrogen peroxide was also up-regulated. Under drought stress, both GSH and AsA are the main antioxidants that play a major role in scavenging ROS.

The *L*. *olgensis* seedlings also responded positively to drought stress through antioxidant metabolism. GSH can increase the expression level of the upstream metabolite directly related to GSH by increasing the expression of the leucyl aminopeptidase gene. Additionally, 5-L-glutamyl-L-alanine, a downstream metabolite of GSH, was detected and down-regulated during drought stress. Thus, both effects combined, the expression level increase of upstream metabolites directly related to GSH and the expression level decrease of downstream metabolites, elevated the synthesis yield of GSH and decreased its degradation rate, which was responsible for maintaining a high intracellular GSH concentration. In the scavenging of ROS, the increased activity of GR and GPX ensured that the conversion of GSSG and GSH was accomplished using the reduced hydrogen provided by glucose-6-phosphate 1-dehydrogenase and 6-phosphate dehydrogenase involved in the conversion of NADP/NADPH, thereby achieving the clearing of active oxygen. In addition, under drought stress, GSH metabolism was actively regulating the expression of GST-related enzymes, which improved the ability for scavenging ROS. Therefore, the regulation of the differential expression of key enzyme genes in GSH metabolism provides drought tolerance in larch trees.

Raffinose series oligosaccharides act as osmotic adjustment substances to stabilize the photosynthetic system under abiotic stress and protect cells as protective agents [33]. In addition, raffinose also scavenges free radicals. UDP-galactose synthesizes inositol galactoside involving the activity of 3-α-galactosyltransferase [34]. The 3-α-galactosyltransferase gene was up-regulated after each of the three drought stress periods, and raffinose was synthesized from inositol galactosides by raffinose synthase. Thus, the 3-galactosyltransferase gene was highly expressed in the galactose metabolic pathway to increase the upstream galactose metabolites and reduce the consumption of stachyose synthase and α-galactosidase, all of which maintained a high intracellular concentration of galactose. Hence, raffinose acted as an osmotic regulator substance and maintained the osmotic pressure in plants to persist under drought.

Studies have shown that drought-tolerant soybean varieties have more starch decomposition than drought-sensitive varieties [35]. Our results were consistent with this conclusion, indicating that the drought resistance mechanism in *L*. *olgensis* was similar to that in other plants. The starch content of the treatment group increased rapidly within 24 h and then decreased. The latter effect might have been caused by the decomposition of starch into soluble sugar molecules to counter the pressure of long-term drought stress. Specifically, the decomposition of maltose into glucose by α-glucosidase increased the osmotic potential of plant cells, preventing excessive water loss due to glucose accumulation.

Under drought stress, sucrose is an important soluble sugar in plants, which diminishes the stress-induced damage [36]. Moreover, sucrose directly acts as an osmotic adjustment substance because under the action of sucrose synthase or α-glucosidase, glucose and fructose are produced, which increase the osmotic potential of plant cells. However, in plants, sucrose also acts as a signal substance, regulates transporters, and induces the expression of resistance genes. In addition, sucrose functions as an antioxidant [37]. Sucrose synthase is an extremely important soluble enzyme in sucrose metabolism, which is found in the cytoplasm. Sucrose synthase not only catalyzes the synthesis of sucrose but also catalyzes its decomposition. This dual property of sucrose synthase may play a critical role in sucrose metabolism. UTP-glucose-1-phosphate uridine acyltransferase catalyzes the synthesis of UDP-glucose from α-D-glucose-1P. UDP-glucose is used for sucrose production by sucrose synthase, but sucrose can also be decomposed into UDP-glucose by the same enzyme.

UTP-glucose-1-phosphate uridine acyltransferase was initially up-regulated under drought stress, which increased the UDP-glucose content. During the first 24 h of drought stress, the sucrose content was increased. Sucrose synthase might have been involved in the synthesis of sucrose. After 48 h and 96 h of drought stress, the sucrose content was decreased, and again, sucrose synthase might have been involved in the decomposition of sucrose to produce glucose and fructose. Our observations indicated that sucrose was an important soluble sugar component in the plants during the early drought period. Thus, sucrose limited the damage caused by drought stress and participated in the drought stress response of *L*. *olgensis*. During long-term drought stress, sucrose decomposed into glucose and fructose to adjust the osmotic pressure, which diminished the effects of drought stress (Fig 7).

## Conclusions

The drought transcriptome of this study included a high proportion of transcripts without annotation. Specifically, there was no annotation for 15.7%, 19.0%, and 17.2% of the DEGs detected after 24 h, 48 h, and 96 h of drought stress, respectively (Supporting information: Functional assignments of differentially expressed genes). Furthermore, there were 29.3%, 40.0%, and 29.6% of unmapped data for metabolites detected after 24 h, 48 h, and 96 h of drought stress, respectively (Supporting information: Differentially expressed metabolites). The lack of gene annotations and metabolite identifications limits our current efforts to study the mechanism of drought stress response in *L*. *olgensis*. However, this lack of functional assignments suggests that currently unknown genes and metabolites with significant effects during drought stress will be identified in future studies of the molecular mechanism of drought resistance in *L*. *olgensis*.

## Materials and methods

### Plant materials and stress treatments

Seeds were obtained from the Jixi provenance planted in Qingshan seed orchard located in Qingshan Forest Farm (130°57’ E, 45°41’ N), Linkou, Heilongjiang Province in China, Seed materials were obtained from public land and permission was obtained from the governmental management unit and the acquisition and breeding of plant materials were complied with local institutional and national guidelines. Seedlings were grown in a culture room in State Key Laboratory of Tree Genetics and Breeding (Northeast Forestry University) with a 16 h light/8 h dark cycle, 75% humidity, and 22°C constant temperature. Full, lustrous *L*. *olgensis* seeds were soaked in deionized water for 4–5 days; the water was changed 3–4 times. The soaked seeds were covered with film coating to preserve moisture and sown onto a nutrient soil matrix composed of clay/vermiculite/perlite (5/3/2, v/v/v), which was used for all culture steps throughout the entire study. After 25–30 days, the needles of the larch seedlings were fully extended. Seedlings with robust and uniform growth were transplanted into culture pots (13 cm diameter × 17 cm height) containing equal amounts of substrate and cultured for 12 months.

One-year-old larch seedlings were harvested for drought treatment. The average seedling height was 16.0±0.5 cm. Drought treatment was simulated in healthy seedlings with consistent growth using 25% polyethylene glycol (PEG) 6000. Samples were collected at four time points: 0 h (control), 24 h, 48 h, 96 h. Whole plants were removed from the pot, immediately washed with deionized water, dried with sterile filter paper, wrapped in tin foil, and quickly frozen in liquid nitrogen for storage at -80°C.

### Biochemical parameter analysis

The 25% PEG-simulated drought treatment was applied to stable and healthy seedlings. Samples were collected at four time points (0 h (control), 24 h, 48 h, 96 h) for biochemical analysis. Each sample included three biological replicates, which consisted of 12 seedlings. The soluble protein (SP) content was determined using Coomassie Brilliant Blue G-250 [38]. Commercial kits were used to measure peroxidase (POD) activity (G0107F, Grace Biotechnology Co., Ltd., Suzhou) and malondialdehyde (MDA) content (G0109F, Grace Biotechnology Co., Ltd., Suzhou). Statistical data processing was performed using Excel 2003. Calculations for the analysis of variance (ANOVA) and the least significant difference (LSD) were calculated using the SPSS 19.0 general linear model.

### RNA extraction and analysis

Total RNA was extracted from fresh tissue using the CTAB method [39]. RNA concentration was measured using a NanoDrop 2000 (Thermo). RNA integrity was assessed using the Agilent RNA Nano 6000 Assay Kit of the Agilent 2100 Bioanalyzer system (Agilent Technologies). Aliquots of 1 μg RNA per sample were used as input material for the RNA sample preparations. Sequencing libraries were generated using the NEBNext®Ultra™ RNA Library Prep Kit for Illumina® (NEB) following the manufacturer’s instructions, and index codes were added to attribute sequences to each sample. Briefly, mRNA was purified from total RNA using poly-T oligo-attached magnetic beads. Fragmentation was carried out using divalent cations under elevated temperature in NEBNext First Strand Synthesis Reaction Buffer (5x). The first strand of cDNA was synthesized using a random hexamer primer and M-MuLV reverse transcriptase. The second strand cDNA synthesis was subsequently performed using DNA polymerase I and RNase H. The remaining overhangs were converted into blunt ends through exonuclease/polymerase activities. After adenylation of the 3′-ends of DNA fragments, NEBNext adaptors with hairpin loop structures were ligated to prepare for hybridization. To select cDNA fragments of preferentially 240 bp in length, the library fragments were purified with the AMPure XP system (Beckman Coulter). The size-selected, adaptor-ligated cDNA was mixed with 3 μL USER Enzyme (NEB) and incubated at 37°C for 15 min, followed by 5 min at 95°C before PCR. PCR was performed with Phusion High-Fidelity DNA polymerase, along with universal PCR primers and index (X) primers. The PCR products were purified (AMPure XP system), and the library quality was assessed with the Agilent 2100 Bioanalyzer system. Clustering of the index-coded samples was performed on the cBot Cluster Generation System using the TruSeq PE Cluster Kit v3-cBot-HS (Illumina) according to the manufacturer’s instructions. After cluster generation, the library preparations were sequenced on an Illumina Hiseq 2000 platform, and paired-end reads were generated.

### Data analysis

#### Quality control

Raw data (raw reads) in Fastq format were initially processed using in-house Perl scripts. In this step, clean data (clean reads) were obtained by removing reads containing adapter, reads containing ploy-N, and low-quality reads from raw data. At the same time, Q20, Q30, GC-content, and sequence duplication level of the clean data were calculated. All downstream analyses were based on clean, high-quality data.

#### Transcriptome assembly

The left read files (read1 files) and the right read files (read2 files) from all libraries/samples were pooled into one large left.fq file and one large right.fq file, respectively. Transcriptome assembly was accomplished based on the left.fq and right.fq using Trinity v2.5.1 [40] with 2 as default for min_kmer_cov and default settings for all other parameters.

#### Quantification of gene expression levels

Gene expression levels were estimated for each sample using RSEM v1.2.19 [41]: Clean data sets were mapped back onto the assembled transcriptome, and the read count for each gene was obtained from the mapping results.

#### Differential gene expression analysis

Differential expression analyses of two conditions/groups were performed using the DESeq R package, which provide statistical routines for determining differential expression in digital gene expression data using a model based on the negative binomial distribution. The resulting *P* values were adjusted using the Benjamini-Hochberg method for controlling the false discovery rate. Genes with an adjusted *P* value <0.05 by DESeq were assigned as differentially expressed. Gene ontology (GO) annotations were obtained using Blast2GO (v2.5) based on Unigene NR annotation results. The background set of topGO analysis included all Unigene GO annotations. GO enrichment [42] analysis of differentially expressed genes (DEGs) was implemented by the topGO R package-based Kolmogorov-Smirnov test. We used KOBAS software [43] to test the statistical enrichment of DEGs in the pathways of the Kyoto Encyclopedia of Genes and Genomes (KEGG) database.

#### Metabolite extraction

Samples were thawed at 4°C on ice. Then, 100 μL of each sample was transferred to a microcentrifuge tube, extracted with 300 μL of methanol, and prepared for analysis by adding 20 μL of an internal standard substance. Samples were vortexed for 30 s, ultrasound treated for 10 min (in ice water), and incubated for 1 h at -20°C to precipitate proteins. Then, the samples were centrifuged at 13,000 × g for 15 min at 4°C. The supernatant (200 μL) was transferred to a fresh 2 mL glass vial for ultra-high performance liquid chromatography-quadrupole time-of-flight mass spectrometry (UHPLC/Q-TOF-MS) analysis, and 20 μL was retained from each sample and pooled as quality control samples.

The UHPLC/Q-TOF-MS analyses were performed using a UHPLC system (1290, Agilent Technologies). MS raw data (.d) files were converted to the mzXML format using ProteoWizard and processed using the R package XCMS (version 3.2).

### Statistical analysis

A principal component analysis (PCA) was performed using the Chenomx NMR Suite 7.7 software. The PCA result was used to present the variance in the data matrix. To analyze the correlations between gene functions, we performed a cluster analysis of the gene expression patterns using Cluster 3.0 and the JavaTreeview software. The Venn diagram and expression analysis of different transcripts were performed using R packages.

## Supporting information

S1 File The top 50 differentially expressed genes and their predicted functions. (XLS)

S2 File Functional assignments of differentially expressed genes. (XLS)

S3 File Differentially expressed metabolites. (XLS)

## Abbreviations

AsA: L-ascorbic acid
DEGs: differentially expressed genes
GO: gene ontology
GPX: glutathione peroxidase
GSH: glutathione
GR: glutathione reductase
GSSG: glutathione disulfide
GST: glutathione S-transferase
HSP: heat shock protein
KEGG: Kyoto Encyclopedia of Genes and Genomes
LEA: late embryogenesis abundant
nsLTPs: non-specific lipid transfer proteins
OPLS-DA: orthogonal projections to latent structures discriminant analysis
PCA: principal component analysis
PEG: polyethylene glycol
POD: peroxidase
ROS: reactive oxygen species
SP: soluble protein
UHPLC/Q-TOF-MS: ultra-high performance liquid chromatography-quadrupole time-of-flight mass spectrometry
VIP: variable influence on projection
